# Maf1 regulates dendritic morphogenesis and influences learning and memory

**DOI:** 10.1038/s41419-020-02809-y

**Published:** 2020-07-30

**Authors:** Kui Chen, Liang Zhu, Lin Guo, Yuan-Bo Pan, Dong-Fu Feng

**Affiliations:** 1 Department of Neurosurgery, Shanghai Ninth People’s Hospital, Shanghai Jiao Tong University School of Medicine, Shanghai, China; 2 Neuroscience Division, Department of Anatomy, Histology, and Embryology, Shanghai Jiao Tong University School of Medicine, Shanghai, China; 3 Institute of Traumatic Medicine, Shanghai Jiao Tong University School of Medicine, Shanghai, China

**Keywords:** Developmental neurogenesis, Cellular neuroscience, Hippocampus

## Abstract

Maf1, a general transcriptional regulator and mTOR downstream effector, is highly expressed in the hippocampus and cortex, but the function of Maf1 in neurons is not well elucidated. Here, we first demonstrate that Maf1 plays a central role in the inhibition of dendritic morphogenesis and the growth of dendritic spines both in vitro and in vivo. Furthermore, Maf1 downregulation paradoxically leads to activation of AKT-mTOR signaling, which is mediated by decreased PTEN expression. Moreover, we confirmed that Maf1 could regulate the activity of PTEN promoter by luciferase reporter assay, and proved that Maf1 could bind to the promoter of PTEN by ChIP-PCR experiment. We also demonstrate that expression of Maf1 in the hippocampus affects learning and memory in mice. Taken together, we show for the first time that Maf1 inhibits dendritic morphogenesis and the growth of dendritic spines through AKT-mTOR signaling by increasing PTEN expression.

## Introduction

Dendrites receive synaptic inputs from other neurons through the somato-dendritic compartment via protrusions called spines, dendritic arborization, and spine formation and are, therefore, critical for the function of neurons^1,2^. The proper formation and morphogenesis of dendrites require specific precise control by external cues and intrinsic genetic programs^2^. Transcriptional control of gene expression represents the main mode of cell internal regulation of dendrite growth controlled by transcription factors, and thus, transcription factors have emerged as key players in dendrite growth^3^. In recent years, a number of transcription factors have been discovered to play important roles in promoting dendritic growth at different stages of dendrite morphogenesis^3^. Nonetheless, the transcription factors that negatively regulate dendritic development remain largely unknown.

Maf1, a transcriptional repressor of RNA polymerase (pol)III, was initially identified in yeast^4,5^, has been found to be highly expressed in the brain and enriched in the hippocampus and the cortex^6^. In addition, Maf1 was found to be a mammalian target of rapamycin (mTOR) downstream effector^7^, while the phosphatidylinositol 3-kinase/protein kinase B/mammalian target of rapamycin (PI3K/AKT/mTOR) pathway has been implicated in neurite outgrowth^8^, neuronal growth^9^, survival^10^, synaptic plasticity^11^, and dendritic arbor development^12,13^. In this report, we demonstrate that Maf1 negatively regulates dendritic outgrowth through PI3K-AKT-mTOR signaling in cultured hippocampal neurons. Moreover, we found that Maf1 affects the learning and memory ability of mice by negatively regulating the growth of dendritic spines in vivo.

## Materials and methods

### Animal care and maintenance

ICR mice (National Rodent Laboratory Animal Resources Shanghai Branch, China) were used in this study. All procedures involving animal experiments conducted in this study were approved and monitored by the Animal Care Committee of the Shanghai Jiao Tong University School of Medicine. No manipulations were performed until animals were given 1 week to acclimate. Animals were housed in cages under a 12-h light/dark cycle prior to the experiment, with free access to food pellets and water.

### DNA constructs

The Maf1 gene was amplified from hippocampal cDNA by PCR. Then, the Maf1 gene was digested with the Xbal1/Not1 enzyme and constructed with the pGFP-C-ShLenti vector. The plasmids were packaged into lentiviruses. The viruses were generated by cotransfection of two helper plasmids (pVSVG and pCMV189) into the packaging cell line HEK293T at a ratio of 5:6:6 pGFP-C-ShLenti-Maf1: pVSVG: pCMV189. Viruses were harvested 48 h after transfection by collecting the medium from transfected cells and were stored at −80 °C.

### Viral construct generation and stereotaxic injection

The Maf1 ShRNA sequences were as follows: ShRNA1: TTGGAGAACTCCAGCTTTGAGGCCATCAA; ShRNA2: TCTGCTTAGCTGAGTGTGACATCTACAGC; and scramble ShRNA (ShSCR): GCACTACCAGAGCTAACTCAGATAGTACT. All ShRNAs were constructed in the pGFP-C-ShLenti vector, and the lentivirus was packaged as mentioned above. To confirm the specificity of the observed Maf1 knockdown phenotype in neurons, ShRNA2 and ShSCR were constructed in the pMT vector. The sequence of PTEN ShRNA (AGGTGAAGATATATTCCTCCAA) was designed against mouse PTEN as described previously^14^ constructed in the pLVX-IRES-mCherry vector, and the lentivirus was packaged as mentioned above.

ShRNA1 and Maf1 for mice were also constructed with the pAAV-CMV-td-Tomato-ShRNA vector or the pAAV-CMV-td-Tomato-3Flag vector, respectively. Packages of AAV9-ShRNA viruses were provided by Shanghai Sunbio Medical Biotechnology, Shanghai, China.

For stereotaxic injection, P1 pups were gently anesthetized with ice for 5–10 min and placed on a stereotactic frame. For bilateral hippocampal area injection, P1 pups were randomly subjected to inject 1 µl AAV9-flag-Maf1 or AAV9- ShRNA -Maf1 into the hippocampus (1 mm posterior and 1 mm left to the midline and 1.2 mm deep into the skull surface). Following injection, the needle was kept at the injection point for an additional 10 min. After injection, the pups were collected on a 37 °C warm plate for recovery. After regaining mobility, the injected pups were rolled in home cage bedding and returned to their mother as a group. Six weeks after viral injection, the infected mice were killed and processed for immunostaining, electron microscopy, WB, or the Morris water maze (MWM).

### Cell cultures, transfection, and drug treatment

Primary hippocampal cultures were prepared from embryonic day 18 (E18) mouse brains^15^. Dissociated neurons were seeded onto poly-d-lysine and cultured in neurobasal medium containing a B27 supplement and GlutaMAX at a density of 75,000/well. At 7 days in vitro (DIV), hippocampal neurons were transfected with the indicated plasmids using the calcium phosphate method^16^ for 6–14 days. When possible, cells were stained for the appropriate epitope tags and GFP to confirm cotransfection. However, in the specific phase of verifying that Maf1 regulates dendritic growth of neurons, three plasmids were cotransfected, and pMT-GFP was mixed with two other plasmids at a ratio of 1:2:2. Rapamycin (Cell Signaling Technology) was added 8 h after transfection to a final concentration of 100 nM. For lentivirus-infected neurons, the cells were transduced at the indicated DIV by adding concentrated lentivirus at a multiplicity of infection (m.o.i.) of 0.5 to the growing media.

### Western blotting

For Western blotting (WB), the procedures were performed as previously described^17^. Briefly, cells were homogenized and solubilized at 4 °C for 30 min in lysis buffer (1% CHAPS, 2.7 mM KCl, 137 mM NaCl, 1.4 mM KH2PO4, 4.3 mM Na2HPO4, pH 7.2, 5 mM EGTA, 5 mM EDTA, 1 mM PMSF, 1 mM Na3VO4, 50 mM NaF, and protease inhibitors). Then, lysates were clarified via centrifugation at 4 °C to remove the insoluble deposit, run on 10% polyacrylamide gels, and then transferred to nitrocellulose membranes. Membranes were blocked for 1–2 h in TBST (150 mM NaCl, 10 mM Tris, 0.1% Tween 20, and pH 7.6) containing 10% BSA. The primary antibodies were diluted in blocking buffer and incubated with membrane overnight at 4 °C. After washing with TBST three times, the blots were incubated with HRP-conjugated secondary antibodies for 1 h at room temperature. After washing with TBST three times again, the blots were exposed to enhanced chemiluminescence substrate. Quantification was performed by analyzing the relative density of the exposed film using Image J.

To dissect the CA1 region for western blot, after killing the mice, they were transcardially perfused with 0.9% saline (4 °C) until the blood was flushed out, afterward the brains were quickly removed and immersed in saline, then the hippocampus was taken from the brain and carefully dissect the CA1 region with microdissection equipment under a dissection microscope at 4 °C.

As primary antibodies for WB, we used mouse anti-β-actin (1:5000, Thermo Fisher Scientific, MA5-15739), mouse anti-GAPDH (1:5000, Sigma-Aldrich, G8795), mouse anti-Flag (1:1000, Sigma, A3682), rabbit anti-Maf1 (1:1000, Abcam), anti-p-Akt (1:1000, Cell Signaling Technology, 4060 s), anti-Akt (1:1000, Cell Signaling Technology, 2920 s), p-mTOR (1:1000, Cell Signaling Technology, 5536 s), anti-mTOR (1:1000, Cell Signaling Technology, 2972 s), anti-PTEN (1:1000, Cell Signaling Technology, 9188 s), anti-p-P70S6K(1:1000, Cell Signaling Technology, 9208 s), anti-P70S6K (1:1000, Cell Signaling Technology, 9202 s), anti-p-S6 (1:1000, Cell Signaling Technology, 5364 s), and anti-S6 (1:1000, Cell Signaling Technology, 2217 s).

### Quantitative real-time PCR

Total RNA was extracted from primary hippocampal cultures using TRIZOL (Invitrogen, USA) following the manufacturer’s instructions. cDNA was treated with the cDNA synthesis kit (Thermo Fisher Scientific) according to the standard protocol. RT-qPCR was performed on the MX3000P System (Stratagene) using Brilliant SYBR Green QPCR Master Mix (Stratagene). The primer sequences are: PTEN, forward primer, 5′-TGGGGAAGTAAGGACCAGAG-3′, reverse primer, 5′-GGCAGACCACAAACTGAGGA-3′. β-actin, forward 5′-CATCCGTAAAGACCTCTATGCCAAC-3′, and reverse, 5′-ATGGAGCCACCGATCCACA-3′. Relative amounts of product transcripts were quantified with the comparative threshold cycle method (DDCt), β-actin was used as an endogenous reference control.

### Luciferase assay

The overexpression cell line of Maf1 was constructed by infecting HT22 cells with lentivirus, and then the recombinant plasmid pGFP-C-PTEN was cotransfected with the control plasmid pMT vector, 48 h after transfection, cells were collected and lysed for luciferase assays using a dual luciferase assay kit (Promega, USA) according to the manufacturer’s instructions.

### Chromatin immunoprecipitation

Chromatin immunoprecipitation (ChIP) assay was performed based on the EZ ChIP™ Chromatin Immunoprecipitation Kit (Millipore, USA) according to the manufacturer’s protocol. Independent chip-PCR assay was used to confirm that identified maf1 regulated PTEN, and DNA fragments of immunoprecipitate were pulled down by anti Maf1 antibody (Abcam) or IgG control antibody (Santa Cruz Biotechnology). The enriched DNA was analyzed by real-time PCR. PTEN primer sequences are: forward primer, 5′-ATTCCGCTGCCTCGGCTGCCAG-3′, reverse primer, 5′-GATGGAAATGGCTCTGGACTTG-3′. Primers for pre-tRNALeu are forward, 5′-GACCCAGTGGCCTAATGGATA-3′, and reverse, 5′-TGGCGACCCAGATGGGACTC-3′. Primers for GAPDH are forward, 5′-AGGAGAGTGTTTGTAAGTCTC-3′, and reverse, 5′-GAGGCCTGGCTTGTGTACCGC-3′.

### Immunofluorescence staining

Primary mouse hippocampal neurons were fixed in PBS containing 4% PFA and 4% sucrose for 30 min at 4 °C. For the brain slice immunofluorescence assay, after cardiac perfusion of mice with 4% PFA and fixation with 4% PFA at 4 °C overnight, 30 μm coronal brain slices were generated. After washing with PBS, both the cells and brain slices were incubated for blocking with permeable buffer (0.3% Triton X-100 in PBS) containing 10% donkey serum for 45–60 min at room temperature. The cells were then incubated with the primary antibodies (diluted in blocking buffer) at 4 °C for 24 h. Dual-immunofluorescence experiments were performed using the following primary antibodies: rabbit anti-Maf1 (1:1000 for cell, 1:100 for slice, Abcam), chicken anti-Map2 (1:10,000 for cell, 1:1000 for slice, Aves Labs, MAP), goat anti-Vglut2 ((1:10,000 for cell, 1:1000 for slice, Abcam, 101760), mouse anti-Flag (1:10,000, Sigma, A3682), and cell nuclei stained with DAPI. The sections or cells were washed three times (10 min for each wash) in 1× PBS plus 0.1% Triton X-100 and then incubated with Alexa Fluor secondary antibodies conjugated to Alexa488 and Alexa555 specific for the primary antibody host plus DAPI for 1 h at room temperature. Following incubation with secondary antibodies, sections were washed three times (10 min each) in PBS, and the chamber slides were then mounted with mounting medium and imaged.

### Image analysis and quantification

Confocal images (Leica SP8) were used to analyze single- and double-transfected neurons. Objectives of 20, 40, and 63× were used. Morphometric analysis and quantification were performed as described previously^16^. Briefly, a z-series of 7–12 images with a 0.5 m–1 μm depth interval, each averaged two times, was taken at 1024 × 1024 pixel resolutions. The resultant stack was flattened into a single image using a maximum intensity projection. For fluorescence intensity analysis, the confocal settings were constant for all of the scans. MetaMorph image analysis software (Universal Imaging Corporation, Downingtown, PA) was used to analyze and quantify the whole-cell morphometry. A ×20 objective was used to measure the total dendrite length (TDL). All dendrites of individual neurons were traced, and the number of pixels was automatically counted and converted to micrometers with MetaMorph. For the dendrite tip number, we calculated the tip of all non-axon protrusions over 10 μm. For Sholl analysis, we drew concentric circles of 15 μm in diameter around the cell body and manually counted the number of dendrites passing through each circle. To calculate the size of the cell somatic cells, the cell soma was outlined, and the pixel area in squared micrometers was automatically calculated. For the analysis of dendritic spines, neurons were imaged with Leica SP8 microscope with a ×63 objective and 2 zoom with 1024 × 1024 pixel resolution. To quantify the shape of the spine and to classify the shape of neuronal spines, the procedure described by Zhu XN et al.^17^ was employed. GFP-positive cells, td-Tomato-positive cells, or dual-labeled neurons were assessed.

### Transmission electron microscopy (TEM)

Mice were anesthetized and perfused with 2% PFA/2% glutaraldehyde in 0.1 M phosphate buffer. Brains were dissected, embedded in chicken albumin agar, and cut into 500 μm sections using a motorized vibrating-blade microtome. CA1 hippocampal regions were dissected, cut into 1 mm^2^ pieces, and further post-fixed with 3% EM grade glutaraldehyde. Ultrathin tissue sections were stained with conventional osmium-uranium-lead method containing 1% ethanolic phosphotungstic acid. The CA1 hippocampus was visualized using a Philips CM-10 TEM operating at 80 kV. Sections were placed on a grid, and nonoverlapping randomized photographs (N20/section) were taken throughout the entirety of the CA1 stratum radiatum. Photographs were digitized, and an investigator blinded to the experimental groups quantified synapses manually. The inclusion criteria for excitatory synapses included the presence of pre- and postsynaptic membranes with a discernible synaptic cleft, the presence of asymmetric pre- and postsynaptic densities, and the presence of synaptic vesicles at the presynaptic terminal as described previously. Pre- and postsynaptic densities from quantified synapses were measured for synaptic area and length using ImageJ.

### Morris water maze

Morris water maze test (MWM) was conducted according to our previous study^18^. Briefly, sixth weeks after AAV-9 injection, learning and memory functions were evaluated. The maze consists of a circular black pool with a diameter of 120 cm and a depth of 50 cm. A black platform with a diameter of 6 cm and a height of 30 cm was placed in the northeast quadrant of the pool. The pool was filled with 22 ± 1 °C water, and the platform was hidden 1 cm below the water surface. In each experiment, mice were released from one of four directions (east, south, west, and north) and allowed to swim. Each mouse was allowed to discover the underwater platform for 60 s. When the mouse found the platform, it was allowed to rest for 20 s on the platform. If the mouse failed to find the platform within 60 s, it was guided to the platform and allowed to remain there for 20 s. The mice were placed in a dry cage followed each trial. Each mouse was tested across four trials starting from four different start positions each day for 5 consecutive days. On the 6th day, each mouse was tested in one trial to assess memory function. Mouse movement was recorded using a video tracking system (DigBehv, Jiliang Software Technology Company, Shanghai, China), and the results were collected and calculated for statistical analysis. A total of 48 mice, 12 per group contributed to the MWM test.

### Statistical analysis

Data are represented as mean ± standard error (SEM) from at least three biologic replicates for experiments. Statistical differences were determined by Student’s *t* test for two-group comparisons or ANOVA followed by Dunnett’s test for multiple comparisons among more than two groups.

## Results

### Knockdown of endogenous Maf1 in hippocampal neurons promotes the branching of dendrites and the growth of dendritic spines

Firstly, immunoblotting was used to detect the expression of Maf1 in 12-week-old mice tissues, Maf1 was found expressed in almost all tissues, especially in the brain, spinal cord, eyes, thymus, lung, liver, and spleen (Supplementary Fig. 1a). In brain, Maf1 was found highly expressed in the hippocampus, cortex, corpus callosum, cerebellum, brainstem, and thalamus (Supplementary Fig. 1b), which was also confirmed by immunofluorescence assay (Supplementary Fig. 1c, d). Maf1 was also found coexisted with the neuron dendritic marker Map2 in brain slices and cultured neurons, which indicates that Maf1 is expressed in neuron dendrites (Supplementary Fig. 1e).

**Fig. 1:**
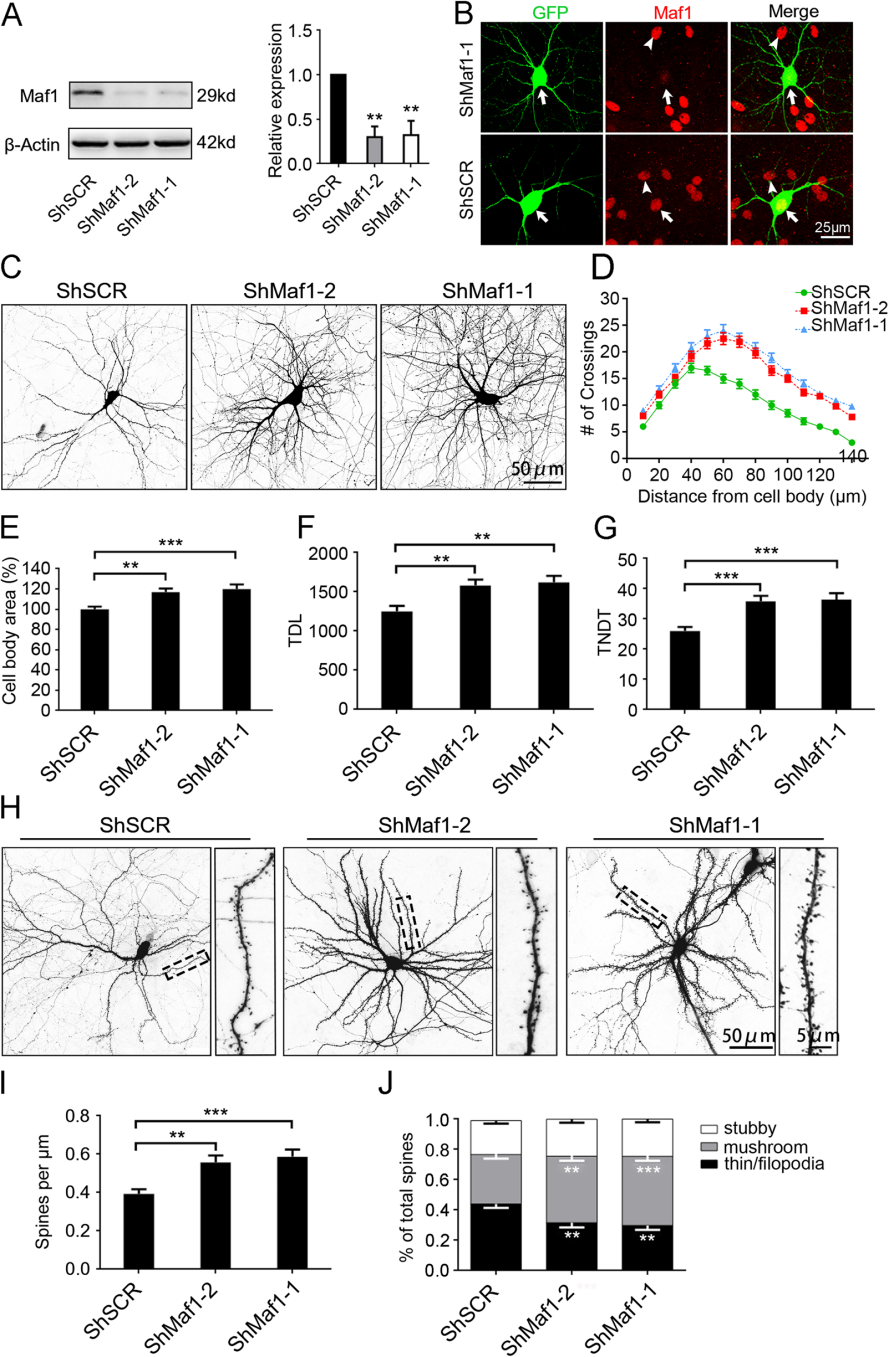
Knockdown of endogenous Maf1 in hippocampal neurons by ShRNAs promotes the branching of dendrites and the growth of dendritic spines. **a** Knockdown of Maf1 by lenti-ShRNA infection of hippocampal neurons cultured in vitro from DIV7 for 6 days was confirmed by immunoblot, right panel shows quantification of WB. **b** Hippocampal neurons cultured in vitro were transfected on DIV7 for 6 days with either scramble ShSCR-GFP or ShRNA against Maf1 (ShMaf1-1-GFP, ShMaf1-2-GFP). Afterward the cells were stained with an antibody against endogenous Maf1, arrow indicates transfected neuron, arrowhead indicates non-transfected cell. **c** Representative images of hippocampal neurons transfected on DIV7 for 7 days with ShSCR-GFP, ShMaf1-1-GFP, or ShMaf1-2-GFP. **d** Sholl analysis of neurons transfected with ShSCR-GFP, ShMaf1-1-GFP, or ShMaf1-2-GFP (ShSCR: *n* = 48; ShMaf1-1: *n* = 48; ShMaf1-2: *n* = 48). **e** Cell body area of hippocampal neurons after Maf1 knockdown (ShSCR: *n* = 48; ShMaf1-1: *n* = 48; ShMaf1-2: *n* = 48). **f**, **g** TDL and TNDT of hippocampal neurons after Maf1 knockdown (ShSCR: *n* = 48; ShMaf1-1: *n* = 48; ShMaf1-2: *n* = 48), respectively. **h** Representative images of hippocampal neurons transfected on DIV7 for 14 days with ShSCR-GFP, ShMaf1-1-GFP or ShMaf1-2-GFP. **i**, **j** are the quantification of dendritic spine densities and the percentages of classification for neurons (ShSCR: *n* = 48; ShMaf1-1: *n* = 48; ShMaf1-2: *n* = 48), respectively. Cell images were obtained from three independent culture batches. Error bars indicate S.E. ***p < 0.001; **p < 0.01.

To investigate the function of endogenous Maf1 in dendrite growth, we targeted Maf1 with two different ShRNAs: ShMaf1-1 and ShMaf1-2. All the ShRNAs have a high knockdown efficiency above 60% knockdown at the protein level (Fig. 1a). ShRNAs directed against Maf1 effectively decreased the level of endogenous protein compared with the un-transfected neurons or in ShSCR-transfected (Fig. 1b). After establishing the effectiveness of ShRNAs tools, we tested the effects of decreased Maf1 activity on dendritic arbor development. Sholl analysis was used to quantify the branching pattern of dendritic trees. In neurons transfected with ShMaf1-1 or ShMaf1-2, the number of crossings reached a peak at 60–65 μm from the cell body, and the number of crossings at 140 μm was still much higher than that in control neurons (Fig. 1c, d). Knockdown of endogenous Maf1 in neurons also leads to a strong increase in cell soma (Fig. 1c, e). In addition, TDL significantly increased under these conditions (by 26% and 29% for ShMaf1-1-GFP, ShMaf1-2-GFP, respectively; Fig. 1f). Finally, transfection with ShMaf1-1 or ShMaf1-2 increased the total number of dendritic tips (TNDT; 38% and 36%, respectively, Fig. 1c, g). Taken together, these results indicate that knocking down Maf1 in neurons promotes the growth of neuron dendrites.

In addition to the significant changes in overall dendritic morphology, knockdown of endogenous Maf1 in neurons also displayed prominent alterations in dendritic spine morphology. 14 days after transfection, knockdown Maf1 increased spine density (by 46% and 41% for ShMaf1-1, ShMaf1-2, respectively; Fig. 1h, i). We also found knockdown Maf1 increased the density of mushroom-shaped spines while reduced the density of filopodia/thin-like protrusions (Fig. 1h, j). There was an increase in mushroom spines by 12.6 and 11.0%, respectively, with a reduction in thin/filopodia-like spines by 12.4 and 14.2%.

### Overexpression of Maf1 in hippocampal neurons suppresses dendrite branching and the growth of dendritic spines in vitro

**Fig. 2:**
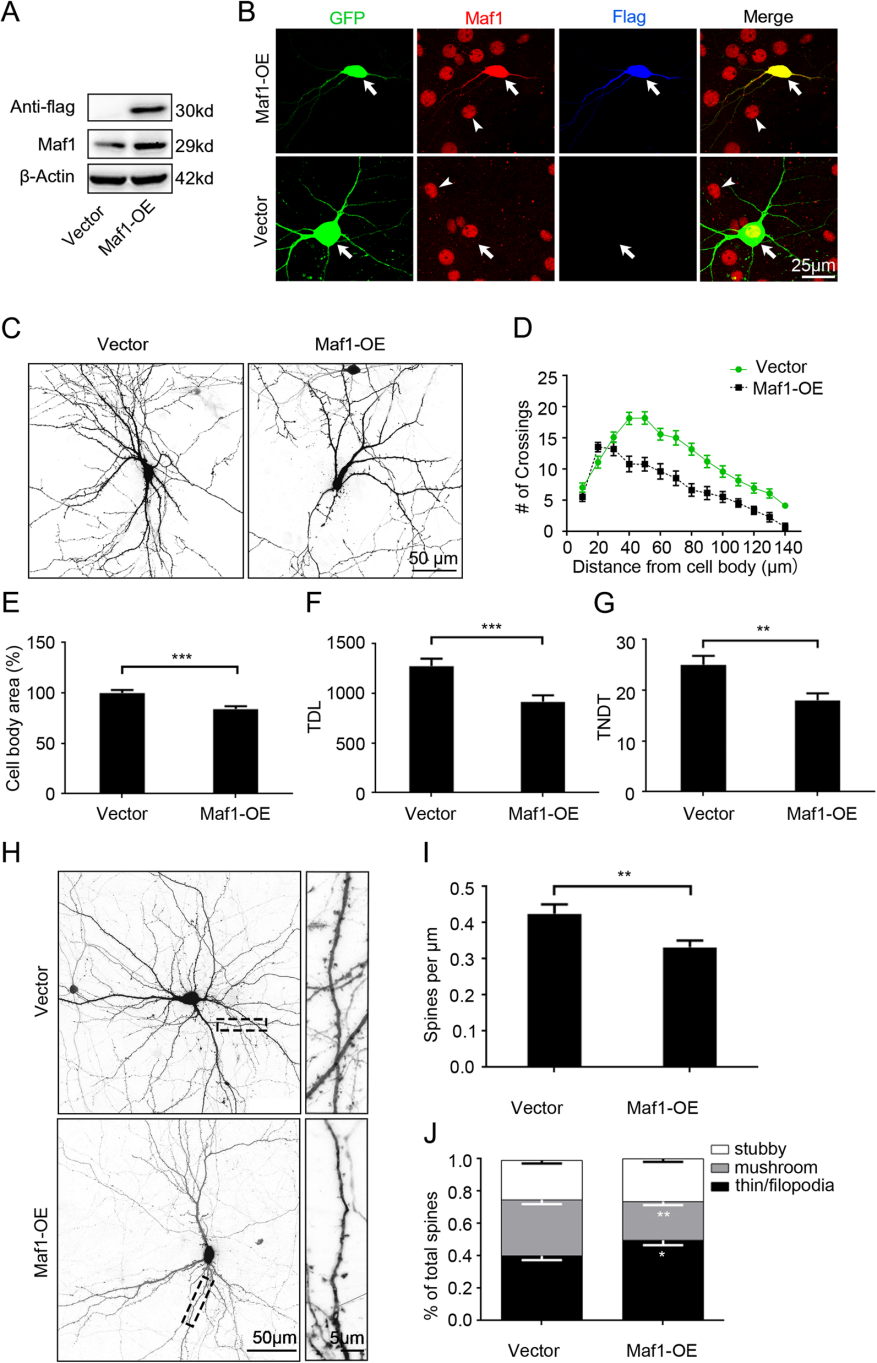
Overexpression of Maf1 in hippocampal neurons suppresses dendrite branching and the growth of dendritic spines. **a** Maf1 overexpression in hippocampal neuron in vitro cultures by lentiviral infection on DIV7 for 6 days was confirmed by immunoblot. **b** Hippocampal neurons cultured in vitro were transfected on DIV7 for 6 days with either control Vector or Maf1-OE-GFP. Afterward, the cells were stained with antibodies against Maf1 and Flag, arrow indicates transfected neuron, arrowhead indicates non-transfected cell. **c** Representative images of hippocampal neurons transfected on DIV7 for 7 days with Vector-GFP or Maf1-OE-GFP. **d** Sholl analysis of neurons transfected with Vector or Maf1-OE (Vector: *n* = 48; Maf1-OE: *n* = 48). **e**–**g** Cell body area, TDL, and TNDT of hippocampal neurons transfected with Vector or Maf1-OE (Vector: *n* = 48; Maf1-OE: *n* = 48), respectively. **h** Representative images of hippocampal neurons transfected on DIV7 for 14 days with Vector or Maf1-OE. **i**, **j** show the quantification of dendritic spine densities and percentages of classification of neurons (Vector: *n* = 48; Maf1-OE: *n* = 48), respectively. Cell images were obtained from three independent culture batches. Error bars indicate S.E. ***p < 0.001; **p < 0.01.

As knockdown of endogenous Maf1 in hippocampal neurons promotes the branching of dendrites and the growth of dendritic spines, we speculate that Maf1 plays a negative regulatory role in the process of neuron dendritic growth. To test this hypothesis, we overexpressed Maf1 in cultured hippocampal neurons. The overexpression of Maf1 was confirmed by WB and immunofluorescence (Fig. 2a, b). As shown in Fig. 2b, staining for Maf1 was significantly increased compared with the un-transfected cells or in Vector-transfected control. In addition, staining for Flag was only shown in Maf1-OE transfected cells.

In contrast to the effect of knocking down Maf1 in neurons, overexpression of Maf1 in neurons suppresses dendrite branching (Fig. 2c). Sholl analysis showed the “peak” of branching in Maf1-OE shifted to the left (Fig. 2d). Maf1 also inhibited cell growth effects, as measured by quantification of the cell body area (Fig. 2e). In addition, the Maf1-OE neurons showed an ~28% decrease in TDL and an ~29% decrease in TNDT (Fig. 2c, f, g). Taken together, these results indicate that Maf1 in neurons can suppress the growth of neuron dendrites.

Maf1 overexpression also suppressed the growth of dendritic spines. In all, 14 days after transfection with Maf1-OE, neurons showed an ~20% decrease in the density of spines (Fig. 2h, i), which also decreased the density of mushroom-shaped spines but increased the density of filopodia/thin-like protrusions. There was a significant decrease in mushroom spines by 10.2%, with an increase in thin/filopodia-like spines by 7.7% (Fig. 2h, j).

### The dendritic growth effects promoted by silencing Maf1 are rescued by overexpression of Rat Maf1

**Fig. 3:**
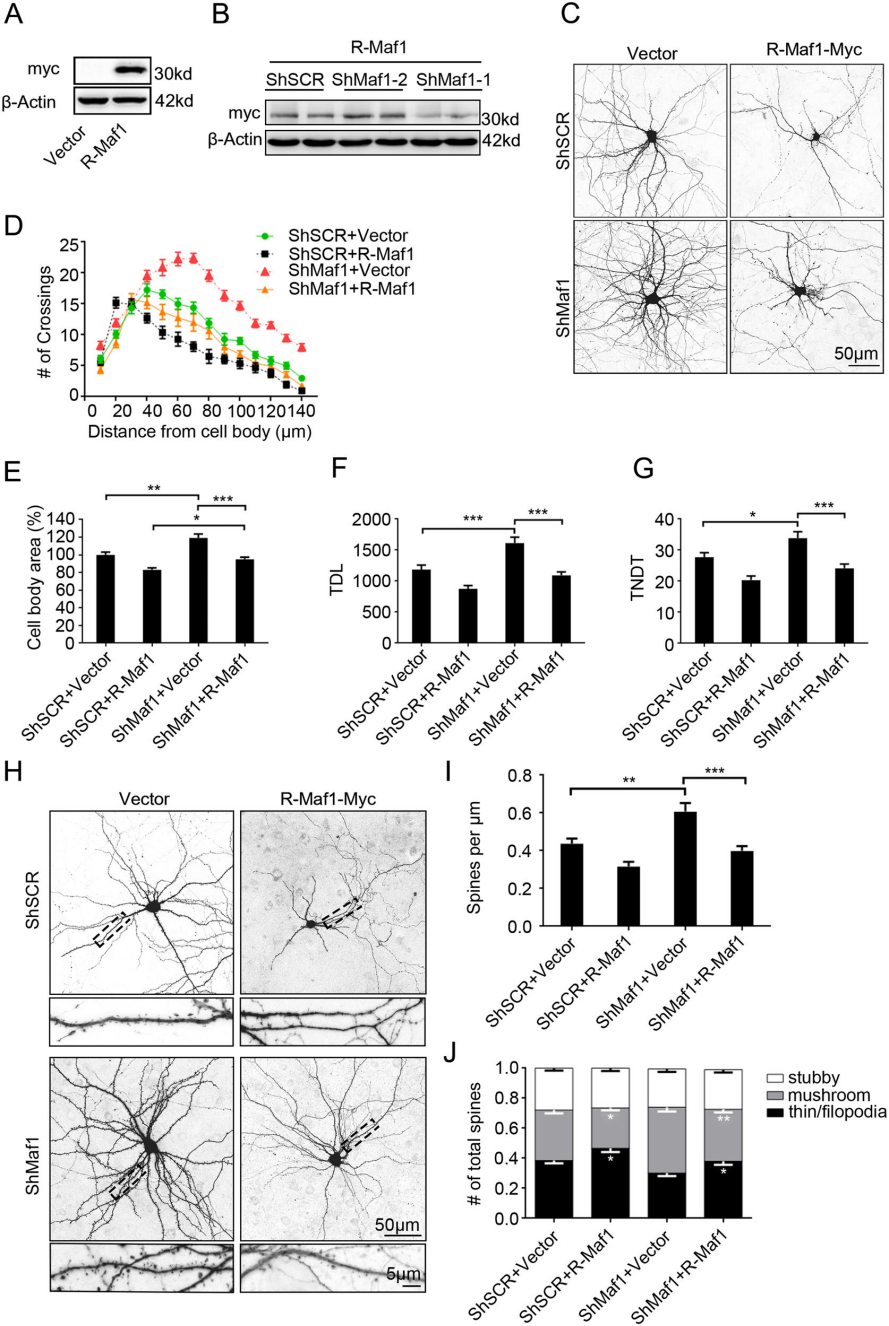
The effects of silencing Maf1 on promoting dendritic spine growth are rescued by overexpression of rat Maf1. **a** Overexpression of Rat-Maf1 was confirmed by immunoblot in HEK293 cells. **b** When overexpressed in HEK293 cells, myc-tagged R-Maf1 was not recognized by cotransfected ShRNAs against mouse Maf1. **c** Hippocampal neurons cultured in vitro were cotransfected on DIV7 for 7 days with Vector (control) or R-Maf1 and scramble ShSCR or ShMaf1. **d**–**g** Neuronal morphology was visualized by cotransfected monomeric green fluorescent protein. Sholl analysis (ShSCR/Vector, *n* = 40; ShSCR/R-Maf1, *n* = 40; ShMaf1/Vector, *n* = 40, and ShMaf1/R-Maf1, *n* = 40) (**d**), cell body area (**e**) (*n* as in **c**), TDL (*n* as in **c**) (**f**), and TNDT (*n* as in **c**) (**g**) of hippocampal neurons after transfection with the indicated plasmids. **h** Representative images of hippocampal neurons transfected on DIV7 for 14 days with vector (control) or R-Maf1 and scramble ShSCR or ShMaf1. **i**, **j** Neuronal morphology was visualized by cotransfected monomeric green fluorescent protein. **i**, **j** show the quantification of dendritic spine densities and the percentages of classification of neurons (ShSCR/Vector, *n* = 48; ShSCR/R-Maf1, *n* = 48; ShMaf1/Vector, *n* = 48, ShMaf1/R-Maf1, *n* = 48), respectively. Cell images were obtained from three independent culture batches. Error bars indicate S.E. ***p < 0.001; **p < 0.01; *p < 0.05.

To further confirm the specificity of the observed Maf1 knockdown phenotype in neurons, we performed “rescue” experiments with plasmids encoding rat Maf1 cDNAs that should not be recognized by ShRNAs designed against mouse sequences. First, we transiently expressed Rat-Maf1 (myc-tagged) in HEK293 cells (Fig. 3a). We then cotransfected ShRNAs and Rat-Maf1 in HEK293 cells, only ShMaf1-2 did not silence the expression of myc-tagged R-Maf1, consistent with the high homology of ShMaf1-1-targeted sequences between mouse and rat (Fig. 3b). Therefore, in the “rescue” experimental phase, the ShMaf1-2 plasmid was used, referred to as ShMaf1.

Hippocampal neurons were simultaneously transfected with ShMaf1 and either a plasmid that encoded R-Maf1 (myc-tagged) or a suitable ShRNA vector. Neurons transfected with ShMaf1 and Vector served as an additional control. In all variants, plasmids that encoded GFP were added to the transfection to visualize the morphology of transfected neurons. The simultaneous knockdown of endogenous Maf1 and overexpression of R-Maf1 significantly prevented the phenotypic effects of Maf1 knockdown (Fig. 3c). Sholl analysis revealed that at most measured distances, especially those closer to the cell soma, the number of crossings was significantly decreased in “rescue” variants (ShMaf1+R-Maf1) compared with knockdown variants (ShMaf1+Vector); the “peak” of branching was shifted leftward in the ShMaf1+R-Maf1-transfected cells compared with knockdown variants (Fig. 3d). In ShMaf1-trasfected cells, R-Maf1 overexpression significantly decreased cell body area (Fig. 3e). Nevertheless, the cell body area did not reach the values of ShSCR/R-Maf1-transfected cells (Fig. 3e). With Maf1 knockdown, the effect of introducing ShMaf1 on TDL (29% increase) was fully restored by the simultaneous overexpression of R-Maf1 (Fig. 3c, f). Similarly, the level of TNDT was highly rescued (Fig. 3f).

In addition, we found that silencing Maf1 promoted dendritic spine growth effects and could also be rescued by overexpression of Rat Maf1 (Fig. 3h–j). In all, 14 days after transfection on DIV7, ShSCR/R-Maf1 showed a significant increase in the number of filopodia/thin-like protrusions with a concomitant significant reduction in the density of mushroom-shaped spine, which was consistent with the effect of overexpression Maf1, while ShMaf1/Vector-transfected neurons showed a significant increase in the density of mushroom-shaped spines. However, the simultaneous knockdown of Maf1 and overexpression of R-Maf1 significantly prevented the phenotypic effects of Maf1 knockdown. For ShMaf1-trasfected neurons, R-Maf1 overexpression significantly decreased the density of spines (from 33% for ShMaf1/Vector to −13% for ShMaf1/R-Maf1 compared with ShSCR/Vector, Fig. 3i). We also found that neurons transfected with ShSCR/R-Maf1 showed a significant increase in the density of filopodia/thin-like protrusions with a concomitant significant reduction in the density of mushroom-shaped spines compared with knockdown variants (Fig. 3j).

### Maf1 negatively regulates AKT-mTOR signaling in the dendritic growth of hippocampal neurons

**Fig. 4:**
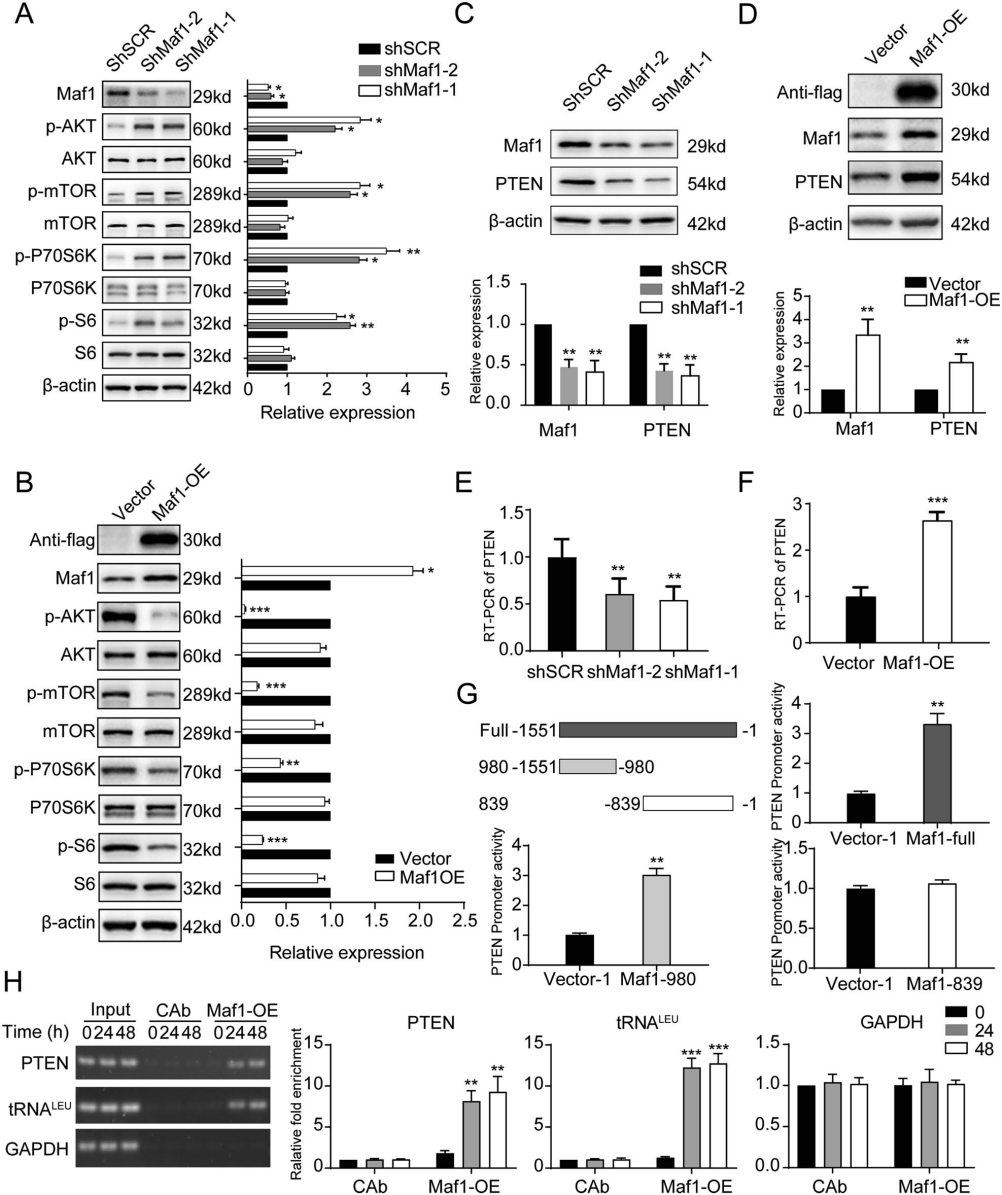
Maf1 negatively regulates AKT-mTOR signaling in the dendritic growth of hippocampal neurons. **a** Hippocampal neurons cultured in vitro were infected on DIV7 for 6 days with either lenti-scramble ShSCR or lenti-ShRNA against Maf1 (ShMaf1-1 and ShMaf1-2). The change in AKT-mTOR signaling following knockdown of endogenous Maf1 in hippocampal neurons was assessed by WB, right panel shows quantification of WB. **b** Hippocampal neurons cultured in vitro were infected on DIV7 for 6 days with either lenti-control vector or lenti-Maf1-OE, WB to ascertain the change in AKT-mTOR signaling following overexpression Maf1 in hippocampal neurons, right panel shows quantification of WB. **c**, **d** The expression of PTEN followed knockdown or overexpression Maf1 in neurons was detected by WB, lower panel shows quantification of WB. **e**, **f** mRNA levels of PTEN was assessed by qPCR following knockdown or overexpression Maf1 in hippocampal neurons. **g** Detection of PTEN promoter activity after overexpression Maf1 in HT22 cells by dual-luciferase reporter assay. Cells were measured for luciferase activity 48 h after transfection. The left upper panel shows the fusion of the luciferase reporter gene to different regions of the PTEN promoter. The left lower panel and the both right panels shows the activity of different luciferase reporters following overexpression Maf1 or not. Data represent mean ± SEM (*n* = 3); **P < 0.01. **h** Maf1 binds to the PTEN promoter. ChIP was performed to detect the degree of Maf1 occupation in the regulatory regions of the PTEN, tRNA^LEU^, and GAPDH promoters at different times (hours). The right panel shows the PCR quantification results. tRNA^LEU^ and GAPDH genes were used as positive and negative controls respectively. Error bars indicate S.E. ***p < 0.001; **p < 0.01; *p < 0.05.

As Maf1 is a major effector of PI3K-Akt signaling, we speculated that Maf1 may regulate dendritic growth by regulating the PI3K/AKT signaling pathway. Indeed, Maf1 knockdown increased phosphorylation of AKT, mTOR, P70S6K, and S6 in neurons, indicating the activation of AKT-mTOR signaling (Fig. 4a). Conversely, Maf1 overexpression reduced AKT-mTOR signaling (Fig. 4b). Maf1 knockdown also reduced PTEN protein (Fig. 4c) and mRNA (Fig. 4e) in neurons, while Maf1 overexpression increased PTEN protein (Fig. 4d) and mRNA (Fig. 4f). These results indicate that Maf1 may be downregulated at posttranscriptional level of PTEN in neurons. In order to clarify how Maf1 regulates the expression of PTEN, we performed the luciferase reporter assay in HT22 cells. As the result showed that overexpression of Maf1 resulted in a significant increase in the activity of the full-length PTEN promoter (−1551 to −1 base pair [bp]; Fig. 4g). Further analysis showed that the Maf1 reaction region is located between −1551 and −980 bp of the PTEN promoter, rather than between −839 and 0 bp (Fig. 4g). ChIP assays confirmed that Maf1 were recruited to the promoter regions of PTEN and tRNA^LEU^, but not the promoter of glyceraldehyde 3-phosphate dehydrogenase (GAPDH; Fig. 4h), which demonstrates the specificity of the ChIP assay. Taken together, these results indicate that Maf1 acted as a transcription activator to enhance PTEN promoter activity and further negatively regulate PI3K/AKT/mTOR signaling.

### Knockdown of Maf1 promoting dendritic growth can be rescued by rapamycin

**Fig. 5:**
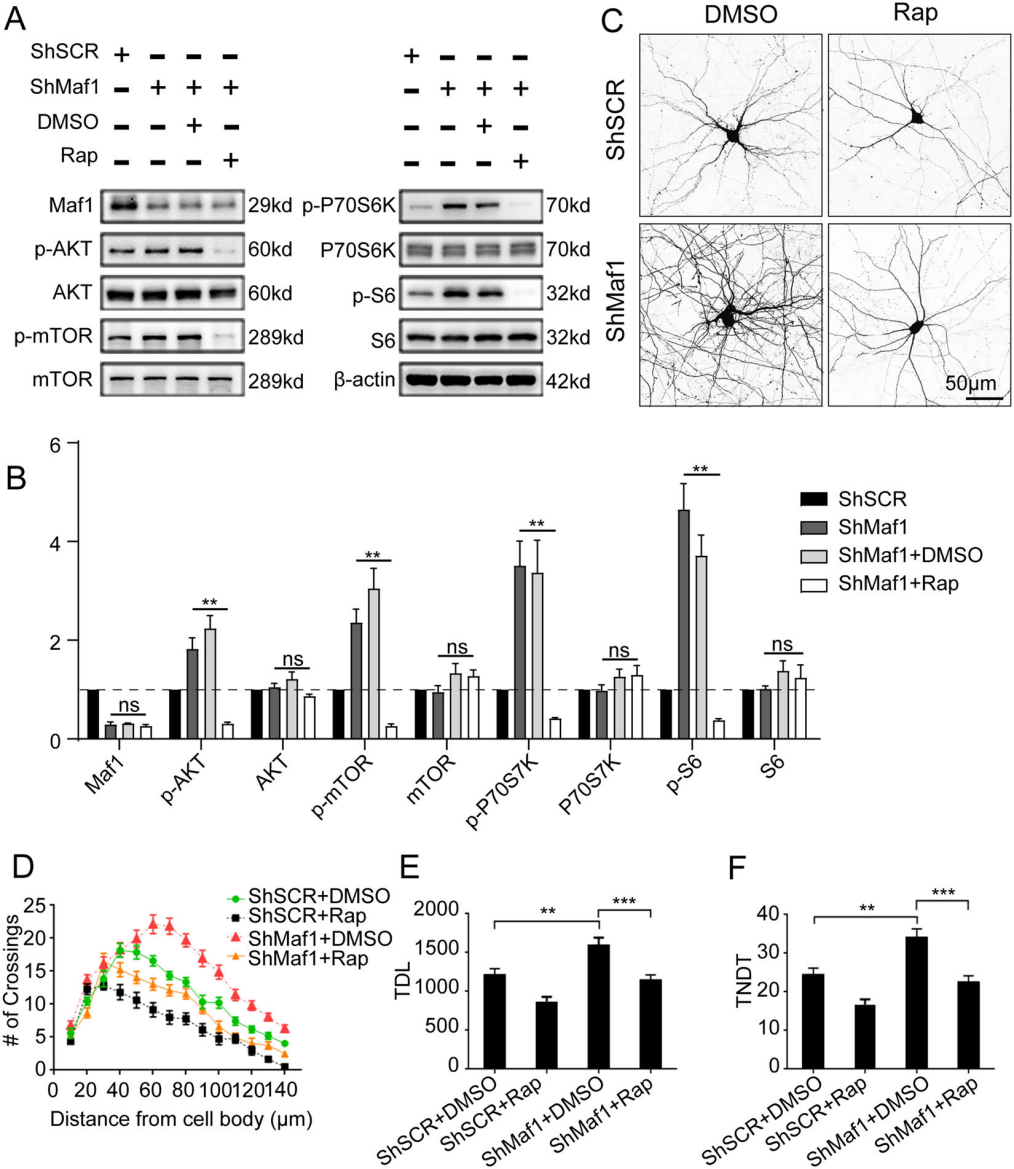
Knockdown of Maf1 promoting dendritic growth can be rescued by rapamycin. **a** The expression of AKT-mTOR signaling in hippocampal neurons infected with lenti-ShSCR or lenti-ShMaf1 at 6 DIV and treated with 100 nM rapamycin over the following 7 days. **b** Quantification of WB in **a**. Data represent mean ± SEM (*n* = 3); ShMaf1+Rap VS ShMaf1+DAMSO or ShMaf1, **P < 0.01. **c** Representative micrographs of hippocampal neurons treated with vehicle or 100 nM rapamycin for 7 days. Neurons were transfected with scramble plasmid of ShSCR or ShRNA against Maf1 (ShMaf1) 1 day before rapamycin treatment. Sholl analysis (ShSCR/DMSO, *n* = 40; ShSCR/Rap, *n* = 40; ShMaf1/DMSO, *n* = 40, ShMaf1/Rap, *n* = 40) (**c**), TDL (*n* as in **c**) (**e**), and TNDT (*n* as in **c**) (**f**) of hippocampal neurons after treatment with the indicated method in **c**. Error bars indicate S.E. ***p < 0.001; **p < 0.01.

To test whether Maf1 regulates dendritic complexity through mTOR, we used a specific inhibitor of mTOR, rapamycin. Neurons were infected by lenti-ShSCR or lenti-ShMaf1 at 6 DIV and treated with 100 nM rapamycin over the following 7 days. This treatment was enough to prevent the phenotypic effects of the Maf1 knockdown, which decreased the phosphorylation of AKT/mTOR signaling (Fig. 5a, b). In ShSCR-transfected cells, rapamycin decreased the number of crossings at all measured points beyond 30 μm (Fig. 5c, d). Rapamycin also decreased TDL in ShSCR-transfected cells by ~28% and TNDT by ~32% compared with control cells transfected with ShSCR/DMSO (Fig. 5c, e, f). Furthermore, rapamycin shifted the Sholl plots of neurons with knockdown of Maf1 back to the control size and shape (Fig. 5c, d), and rapamycin abolished the increase in TDL and TNDT induced by knockdown of Maf1 in neurons (Fig. 5e, f). Taken together, rapamycin simplified dendritic arbor morphology and abolished the phenotypic effects of Maf1 knockdown in neurons.

### Overexpression of Maf1 suppressing dendritic growth can be partially rescued by knockdown PTEN

**Fig. 6:**
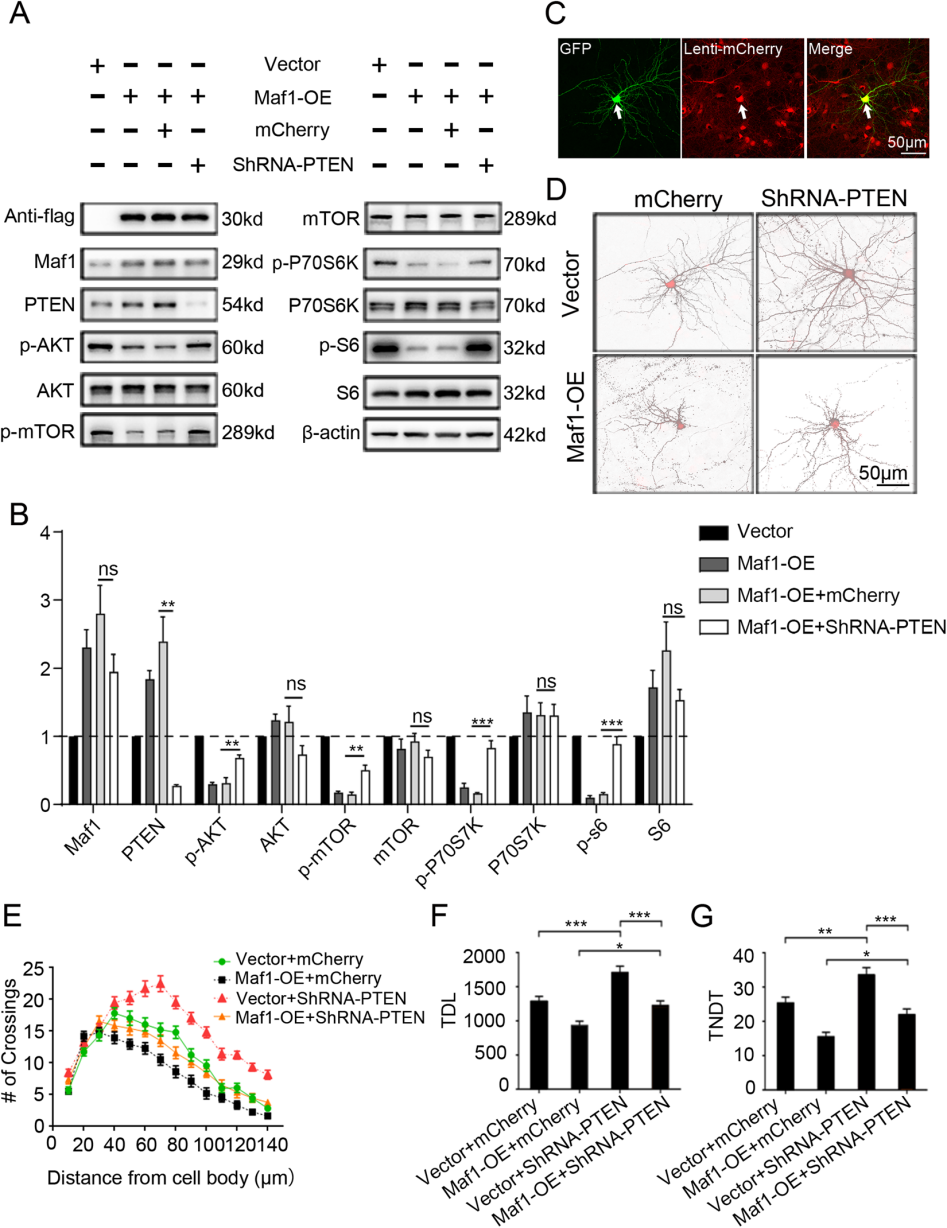
Overexpression of Maf1 suppressing dendritic growth can be partially rescued by knockdown PTEN. **a** The expression of AKT-mTOR signaling in hippocampal neurons infected with lenti-ShSCR or lenti-ShMaf1 and lenti-ShRNA-PTEN-mCherry or control vector lenti-mCherry at 6 DIV for 7 days. **b** Quantification of WB in **a**. Data represent mean ± SEM (*n* = 3); Maf1-OE+mCherry VS Maf1-OE+ShRNA-PTEN, ***p < 0.001; **p < 0.01. **c** Representative micrographs of neurons transfected with GFP-enriched plasmids were colocalized with the lentivirus-infected neurons with mCherry. **d** Representative micrographs of neurons transfected with GFP-enriched plasmids of control vector or Maf1-OE at 6 DIV and then treated with lenti-ShRNA-PTEN-mCherry or control vector lenti-mCherry 2 days later. Sholl analysis (Vector/mCherry, *n* = 40; Vector/ShRNA-PTEN-mCherry, *n* = 40; ShMaf1/mCherry, *n* = 40, ShMaf1/ShRNA-PTEN-mCherry, *n* = 40) (**e**), TDL (*n* as in **e**) (**f**), and TNDT (*n* as in **e**) **g** Hippocampal neurons after treatment with the indicated method in h. Cell images were obtained from three independent culture batches. Error bars indicate S.E. ***p < 0.001; **p < 0.01; *p < 0.05.

To determine whether altered PTEN expression plays a role in Maf1 suppression of dendrite branching, we used short hairpin RNA (ShRNA) to knockdown PTEN in Maf1-overexpressing neurons. PTEN knockdown blunts the ability of Maf1 overexpression to suppress phosphorylation of AKT, mTOR, P70S6K, and S6 (Fig. 6a, b). Then we transfected DIV6 neurons with Vector or Maf1-OE and treated them with lenti-ShRNA-PTEN-mCherry or control vector lenti-mCherry 2 days later, some of the neurons transfected with GFP-enriched plasmids were colocalized with the lentivirus-infected neurons with mCherry were found (Fig. 6c). PTEN knockdown in neurons increased the number of crossings at all measured points beyond 20 μm, and the number of crossings at 140 μm was still much higher than that in the Vector/mCherry neurons (Fig. 6d, e). PTEN knockdown also increased the TDL in Vector by ~33% and the TNDT by ~32% compared with control with Vector/mCherry (Fig. 6d, f, g). On the other hand, PTEN knockdown partially inactivated the ability of MAF1-OE to inhibit dendritic branching. Sholl analysis showed that compared with Maf1-OE/lenti-Vector-mCherry, the number of crossings at the maximum was significantly different, and the “peak” of branching was shifted rightward in Maf1-OE/lenti-ShRNA-PTEN-mCherry (Fig. 6d, e). In Maf1-OE transfected neurons, PTEN knockdown increased TDL by ~31% and TNDT by ~31% (Fig. 6d, f, g). These observations indicate that Maf1 positively regulates PTEN expression, which is important for Maf1 to regulate dendritic growth of hippocampal neurons.

### Maf1 negatively regulates the growth of dendritic spines in vivo

To further investigate the function of Maf1 on neuron dendrites in vivo, we utilized rAAV-td-Tomato-mediated delivery. At postnatal day 1, rAAV was injected into the CA1 area with stereotactic microinjections. A total of 12 mice, 3 mice per group contributed for stereotaxic injection. Six weeks later, the CA1 hippocampus area was infected with the AAV virus, as indicated by red fluorescence (Supplementary Fig. 2a). The expression of Maf1 following AAV-td-Tomato injection was then confirmed by WB, the Maf1 protein expression was significantly increased in rAAV-Maf1-OE compared with rAAV-Vector-td-Tomato (Supplementary Fig. 2b, c), while the expression of Maf1 was suppressed in rAAV-ShMaf1 but not with rAAV-ShSCR.

**Fig. 7:**
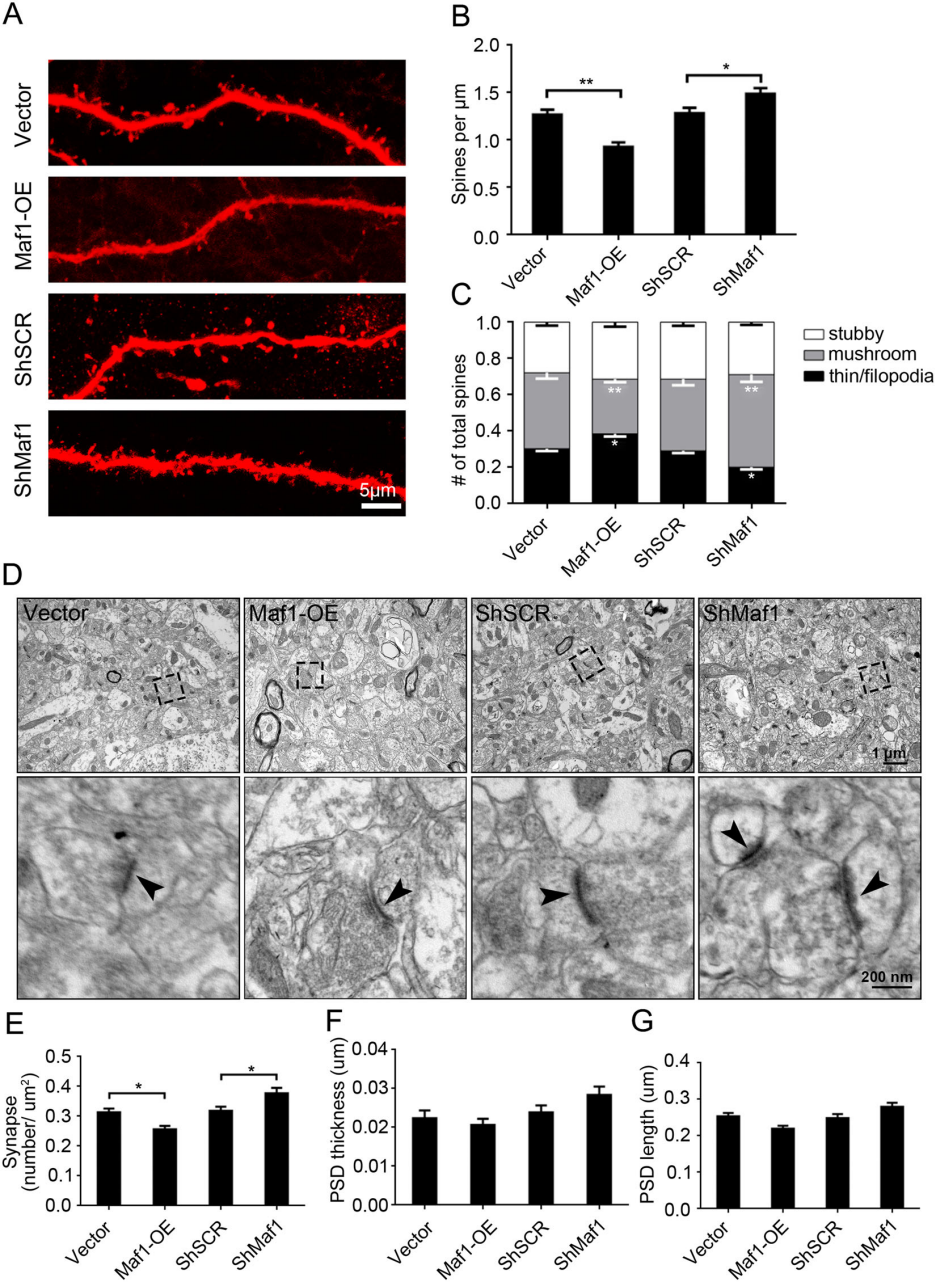
Maf1 negatively regulates the growth of dendritic spines in vivo. **a** Representative images of dendrite spines in CA1 stratum radiatum hippocampal neurons following rAAV injection for 6 weeks with Vector, Maf1-OE ShSCR, or ShMaf1. **b**, **c** are the quantification of dendritic spine densities and percentages of classification for neurons (Vector: *n* = 48; Maf1-OE: *n* = 48; ShSCR: *n* = 48; ShMaf1: *n* = 48), respectively. **d** Electron micrographs showing representative synapses of rAAV-Vector, Maf1-OE ShSCR or ShMaf1 neurons. **e** Quantification of synapses showed that overexpression of Maf1 in the hippocampus resulted in a reduction in synaptic number, while knockdown of Maf1 increased synaptic number in the hippocampus. **f**, **g** Quantification of PSD thickness and length showed overexpression or knockdown of Maf1 in the hippocampus did not change the length or thickness of PSDs. All data are presented as the mean ± SEM with *n* = 4/group and at least 20 micrographs per animal. ***p < 0.001; **p < 0.01; *p < 0.05.

Six weeks after rAAV-td-Tomato injection, rAAV-Maf1-OE showed a significant decrease in spine density by 26% compared with rAAV-Vector, while rAAV-ShMaf1 showed a ~16% increase in the density of spines compared with rAAV-ShSCR (Fig. 7a, b). Consistent with in vitro studies, we also found that overexpression of Maf1 reduced the density of mushroom-shaped spines and increased the density of filopodia/thin-like protrusions, while knockdown of Maf1 by rAAV increased the density of mushroom-shaped spines and decreased the density of filopodia/thin-like protrusions in vivo (Fig. 7c).

To evaluate whether the changed spines are associated with synapses, we examined the synaptic ultrastructure in the CA1 of the hippocampus at 6 weeks following rAAV injection (Fig. 7d–g). In all, 10 regions of interest from four nonadjacent sections in each CA1 stratum radiatum from the hippocampus were imaged to quantify synaptic structures. Asymmetric synapses were quantified if there was a presynaptic terminal containing synaptic vesicles, a submembrane postsynaptic density (PSD), and a visually discrete synaptic cleft. Mice overexpressing Maf1 showed a significant ~18.5% reduction (*p* < 0.001) in synaptic number compared with vector controls, while Maf1 knockdown in the CA1 stratum radiatum resulted in a significant increase (*p* < 0.001) in synaptic number compared with ShSCR scramble controls, suggesting that Maf1 may regulate synaptic formation or maintenance during development (Fig. 7e). However, there was no change in the length and thickness of PSDs with overexpression or knockdown of Maf1 in the hippocampus.

### Expression of Maf1 in the hippocampus affects learning and memory in mice

To determine the effect of Maf1 expression in the hippocampus on learning and memory behaviors, we subjected mice to a behavioral assay, namely, the fixed platform version of the MWM. At postnatal day 1, we carried out bilateral hippocampal injections of rAAV. 6 weeks later, the mice were subjected to MWM learning acquisition trials for 5 consecutive days. A probe trial that measured the ability of mice to locate the quadrant that previously contained the hidden platform was carried out 24 h after the final acquisition trial on day 6.

**Fig. 8:**
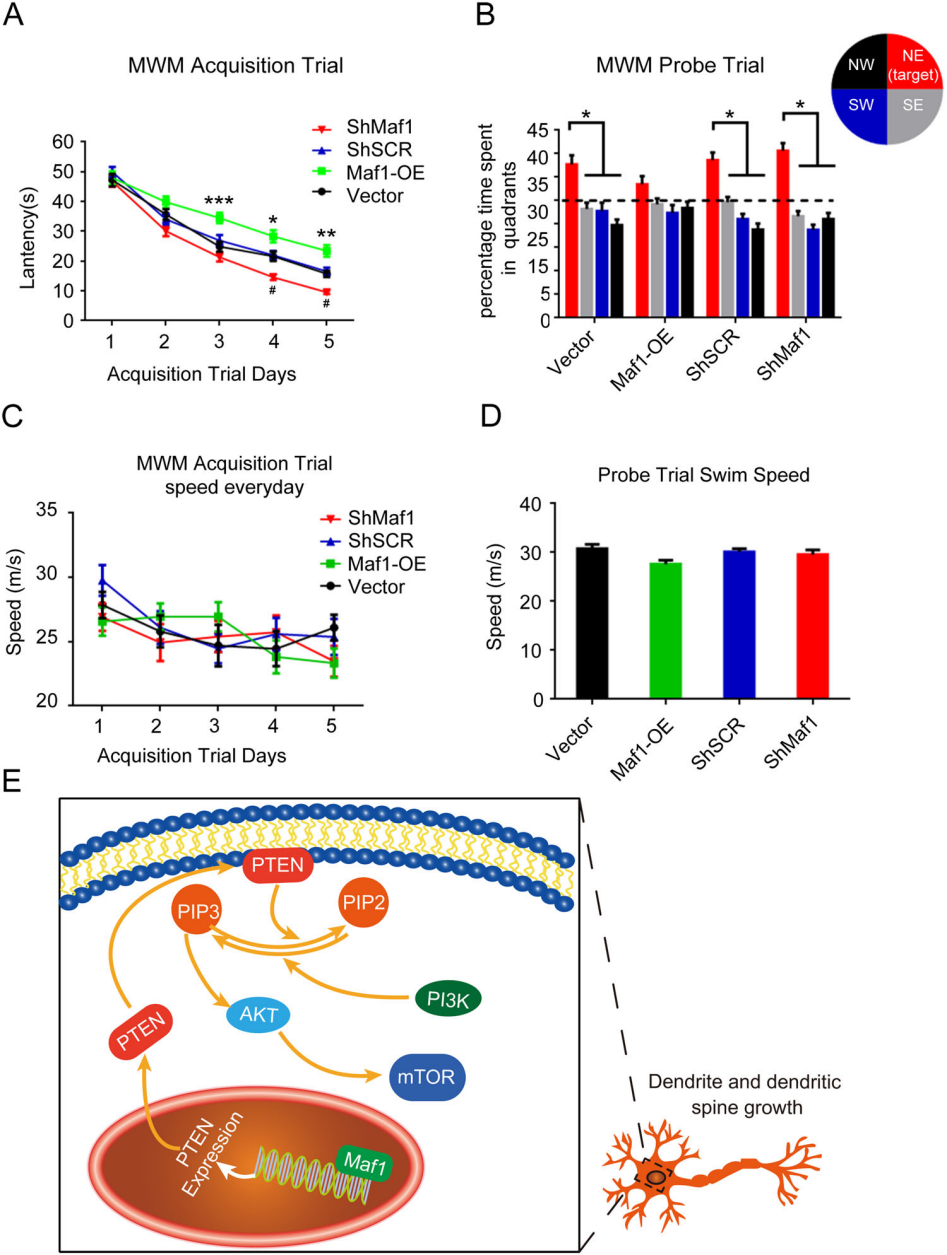
Evaluation of learning (acquisition) and spatial memory retention (probe) using the Morris water maze after overexpression or knockdown Maf1 in the hippocampus. The latency to find the escape platform (**a**) and the time spent in all quadrants (**b**) were measured. **a** The repeated two-way ANOVA showed an increase in the rAAV-Maf1-OE group compared with the rAAV-Vector group (group, *F* (4, 470) = 84.19, *p* < 0.0001; day *F* (1, 470) = 27.56, *p* < 0.0001; interaction *F* (4, 470) = 2.179, *p* = 0.0703), and the Bonferroni post hoc test revealed the significance between groups on days 3–5: while the same method showed a decrease in the rAAV-ShMaf1 group compared with the scramble rAAV-ShSCR group (*F* (4, 470) = 163.5, *p* < 0.0001; day *F* (1, 470) = 22.98, *p* < 0.0001; interaction *F* (4, 470) = 0.5585, *p* = 0.6929), and the Bonferroni post hoc test revealed significance between groups. **b** During the probe trials, the rAAV-Vector, rAAV-ShSCR, and rAAV-ShMaf1 mice demonstrated spatial memory retention, spending a significantly higher percentage of time in the target quadrant (NE) than in all other quadrants (Min test). However, the rAAV-Maf1-OE mice did not show a preference for the target quadrant compared with the other quadrants (Min test). *p < 0.05. The swim speed of mice in all groups on acquisition days 1–5 (**c**) and on the probe test day 6 were measured (**d**). **e** Schematic representation showing Maf1 inhibits dendritic morphogenesis through AKT-mTOR signaling by increasing PTEN expression. The error bars represent the S.E.M. NW northwest, NE northeast, SE southeast, SW southwest. *p < 0.05, **p < 0.01, ***p < 0.001.

All the groups of mice showed daily improvements in the ability to locate the hidden platform during the acquisition trials of the MWM task; however, acquisition deficits were observed in Maf1-OE mice. The average latency to find the hidden platform increased significantly from day 3 in rAAV-Maf1-OE mice relative to Vector control mice. In contrast, knockdown of Maf1 (rAAV-ShMaf1) resulted in reduced latency from day 4 compared with scramble rAAV-ShSCR control (Fig. 8a). During probe trial testing, the different percentages of time spent in quadrants were first analyzed to evaluate the preference of mice for the target quadrant compared with the other three quadrants. We found that mice of the rAAV-Vector, rAAV-ShSCR or rAAV-ShMaf1 groups spent a significantly higher percentage of time in the target quadrant, which had contained the platform during training (*p* < 0.05) than in the other three quadrants (Fig. 8b). However, the rAAV-Maf1-OE mice showed spatial memory impairment, as indicated by the lack of significant differences for preference between the target quadrant and the other quadrants (*p* > 0.1; Fig. 8b). Subsequent analysis was performed to evaluate the distribution of time that the mice spent in the target quadrant (NE) of all groups. We found a significant difference between the rAAV-Vector group and the rAAV-Maf1-OE group (*p* < 0.05). However, compared with rAAV-ShSCR mice, although the distribution of time that rAAV-ShMaf1 mice spent in the quadrants showed a certain increase, there was no significant difference (*p* > 0.05). This result indicates that the capacity to learn the task and memory performance of rAAV-ShMaf1 mice were the same as those of rAAV-ShSCR mice. In addition, we found no evidence that exercise performance was impaired, as swimming speed was unaffected on any day of acquisition testing or the probe trial (Fig. 8c, d). Taken together, these findings demonstrate that Maf1 inhibits dendritic morphogenesis and the growth of dendritic spines through AKT-mTOR signaling by increasing PTEN expression (Fig. 8e).

## Discussion

The development of dendrites and spines in mammals is a complex process^2^. Accumulating evidence highlights the importance of transcription factors in the control of dendrite morphogenesis^2,3,19^. There has been great interest in studying the role of transcription factors as negative regulators of dendritic growth. For instance, MeCP2 is a ubiquitous protein that acts as a global repressor of transcription and was found to be involved in Rett syndrome (RTT)^20,21^, either elimination or overexpression MeCP2 leads to a decrease in the complexity of dendritic arbors^22^; MeCP2 also strongly inhibits dendritic and spine growth, which depends on the interaction of MeCP2 and DiGeorge syndrome critical region 8 (DGCR8)^23^. Moreover, increased global transcriptional repressor element 1 (RE-1) silencing transcription factor (REST/NRSF) activity was found in several neurological diseases, such as Huntington’s disease and cerebral ischemia^24^. Overexpression of REST in primary hippocampal neurons significantly reduced dendritic length and arborization, while knockdown of REST had no difference in dendritic development^25^. In addition, the brain-enriched protein FOXO6 suppressed the growth of dendrites while simultaneously promoting the growth of axons^26^, and the transcription factors Cux1^27^ and Sp4^28^ were also found to have a negative regulatory effect on dendritic morphogenesis. Thus, identifying transcriptional repressors and signaling pathways that control dendrite morphogenesis and connectivity is of great importance for understanding the functions of the mature nervous system.

Maf1, a general transcriptional repressor and mTOR downstream effector, was found to be a negative regulator of Pol-dependent gene transcription^29–31^. Maf1 is well conserved in yeast, worms, fruit flies, plants, and mammals^29^. Previous studies have focused on its role in growth, metabolism, aging, cancer, and so on^32^. Recently, Maf1 was found to be highly expressed in the brain, enriched in the hippocampus and cortex, and highly expressed in the somatic and dendritic cytoplasm^6^. However, the role of Maf1 remains poorly characterized in the nervous system, such as in dendrites.

In this study, using ShRNAs to knock down Maf1 revealed that Maf1 knockdown promotes the branching of dendrites and the growth of dendritic spines (Figs. 1, 7), while Maf1 overexpression suppresses dendrite branching and dendritic spine growth (Figs. 2, 7). Rapid extension and arborization of dendrites occurs during the growth of dendrites^33^, and thus, the ability of Maf1 knockdown to promote the branching of dendrites may result from either the increased formation of new branches or the decelerated retraction of existing branches. Therefore, we revealed a novel role of Maf1 in neurons in that Maf1 may negatively regulate dendrite and spine morphogenesis. However, the mechanism responsible for the effect of Maf1 on dendrite morphology remains to be elucidated.

Palian et al. found that Maf1 is a downstream effector of PTEN/PI3K signaling, which is important for suppressing hepatocarcinogenesis by coregulating lipid metabolism and oncogenesis^34^. Furthermore, expression of Maf1 in PTEN-deficient human glioblastoma cells inhibits anchorage-independent growth^29^. Another study found that Maf1 suppresses hepatocarcinogenesis by suppressing AKT-mTOR signaling through activation of PTEN transcription^35^, which indicated Maf1 could also act as a transcription activator excepted for its canonical role as a transcriptional repressor. More recently, Taxifolin was found to bind with liver-X-receptor (α and β) to attenuate 7,12-dimethylbenz(a)anthracene-induced mammary carcinogenesis through the mTOR/Maf1/PTEN pathway^36^. Therefore, Maf1 is a main effector of PTEN/PI3K signaling. While the PI3K/Akt/mTOR pathway has been implicated in dendritic morphogenesis^16,37^, it is likely that Maf1 mediates its effects on dendrite formation mainly through mTOR. Indeed, we found that Maf1 knockdown increased AKT and mTOR phosphorylation and decreased PTEN expression, while Maf1 overexpression decreased AKT and mTOR phosphorylation and increased PTEN expression (Fig. 4). Given the known role of mTOR in promoting dendritic outgrowth, persistent mTOR activation and decreased PTEN expression due to Maf1 knockdown is a plausible mechanism for dendritic growth. However, Kumar et al.^37^ found that overexpression of constitutively active PI3K or Akt significantly increased the number of filopodia protrusions, accompanied by a significant decrease in the density of mushroom-shaped spines, while chronic inhibition of PI3K/Akt/mTOR signaling by drug treatment reduced both dendritic spines and dendritic filopodia at 15 DIV. In the current study, we found that Maf1 knockdown in neurons increased the density of spines and mushroom-shaped spines, while overexpression of Maf1 reduced both dendritic spines and mushroom-shaped spines at 21 DIV. The difference is believed to be due to the different time points of observation, since 15 DIV is a stage during which dynamic dendritic filopodia are gradually replaced by mushroom-shaped mature spines in neurons^37^.

Rapamycin, a specific inhibitor of mTOR that blocks the effects of PI3K and Akt on dendrite branching^16,37^, was used in this study to test whether Maf1 regulates the growth of dendritic cells through mTOR. We revealed that rapamycin abolished the phenotypic effects of Maf1 knockdown (Fig. 5). In addition, PTEN, a negative regulator of the AKT-mTOR pathway, was found in this study to be positively correlated with the Maf1 level (Fig. 4), which negatively regulates dendrite branching, as PTEN knockdown by siRNA increased the branching of dendrite^16^. We also found that knockdown of PTEN by ShRNA increased the branching of dendrite and partially rescued the phenotypic effects of overexpressing Maf1 in neurons (Fig. 6). These data suggest that Maf1 inhibits dendritic growth mainly by suppressing AKT-mTOR signaling through activation of PTEN.

It is known that mTOR controls cell growth partially through the phosphorylation of the downstream effector p70S6K^38^. Jaworski et al.^16^ showed that p70S6K is indeed crucial for proper dendritic arborization. In this study, we found that Maf1 negatively regulates the expression of P70S6K (Fig. 4), suggesting that Maf1 may regulate translation through the mTOR pathway in the growth of dendrites. S6, the downstream target of p70S6Ks, which is largely controlled by PI3K/Akt/mTOR signaling^37^, was also found in this study to be negatively regulated by Maf1, which further indicated that Maf1 negatively regulates AKT-mTOR signaling in the dendritic growth of hippocampal neurons.

Dendrite branching patterns strongly influence the processing of information across the brain, and defects in dendrite architecture are associated with many neurodegenerative diseases^39^. Thus, a better understanding of the precise regulation of dendritic growth could provide useful insight for these pathological conditions. In this study, we assessed the role of Maf1 in proper hippocampal dendrite growth. In accordance with the stunted dendritic arbors of hippocampal cells observed following knockdown or overexpression of Maf1 in vivo, we found that these animals, especially rAAV-Maf1-OE mice, present behavioral deficits that involve hippocampal circuitry. However, rAAV-ShMaf1 mice only showed a significantly reduced latency from day 4 in the acquisition trials compared with scramble control rAAV-ShSCR mice, while there was no significant difference in the time spent in the quadrants between rAAV-ShMaf1 and rAAV-ShSCR mice, which may be attributed to compensatory effects. As changes in spines and synaptic connections are considered essential for learning and memory formation^40,41^, we speculate that the effects of Maf1 expression on learning and memory in mice may be due to Maf1 influencing the density of dendritic spines and the number of mushroom dendritic spines.

In conclusion, to the best of our knowledge, this is the first study to elucidate the role of Maf1 in dendritic morphogenesis and the growth of dendritic spines. We demonstrated that Maf1 inhibited dendritic morphogenesis and the growth of dendritic spines during neuron development. The function of Maf1 is exerted through AKT-mTOR signaling by regulating PTEN expression in dendrites.

## Supplementary information

Supplementary Figure 1

Supplementary Figure 2

Supplementary Figures Legend
