# Supplementary figures and images for: Maf1 regulates dendritic morphogenesis and influences learning and memory
Source: Cell Death Dis. 2020 Jul 30;11(7):606. doi: 10.1038/s41419-020-02809-y (PMC7393169; doi:10.1038/s41419-020-02809-y)

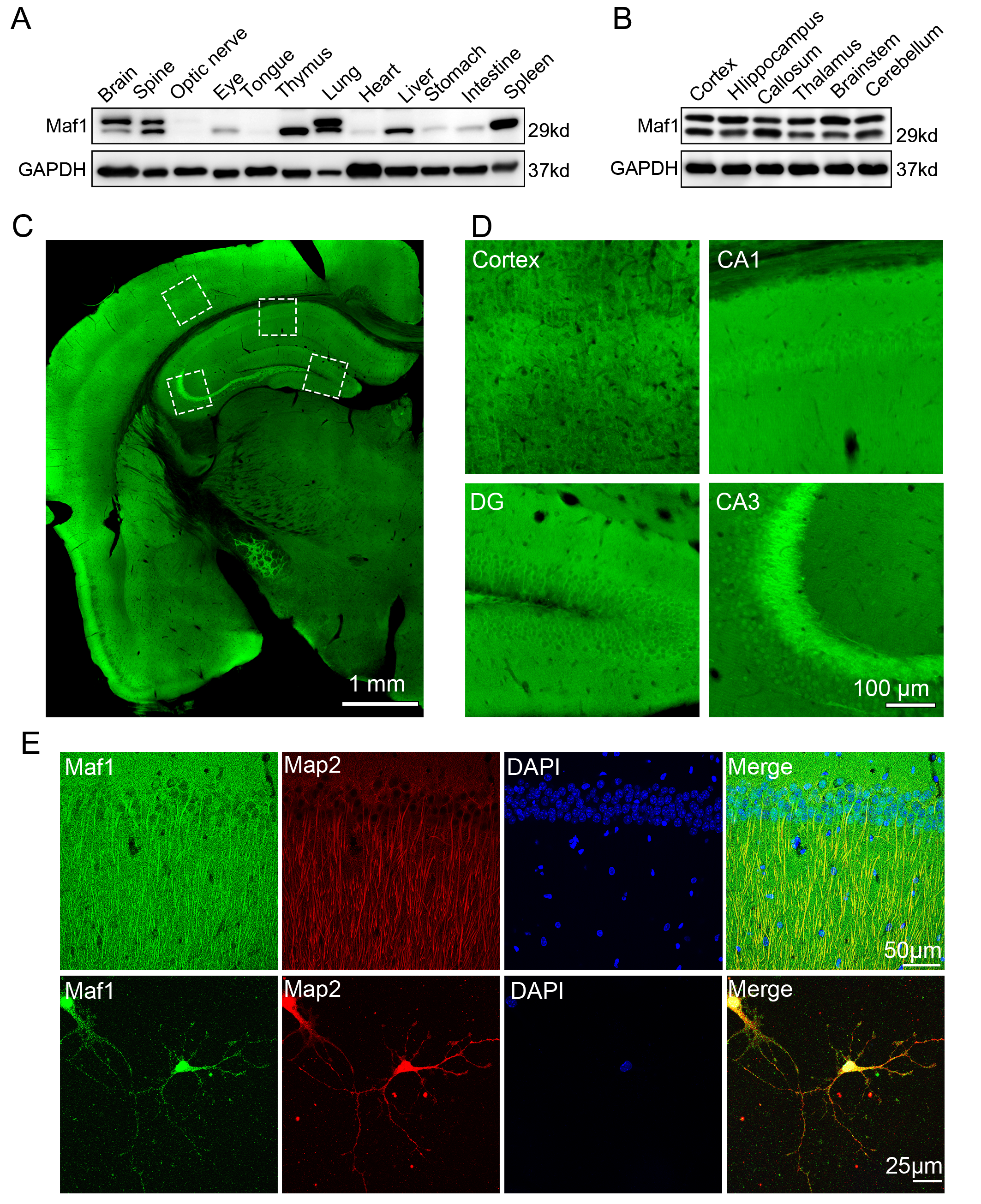

Supplement: Supplementary file 1 — Supplementary Figure 1 [file 41419_2020_2809_MOESM1_ESM.tif]

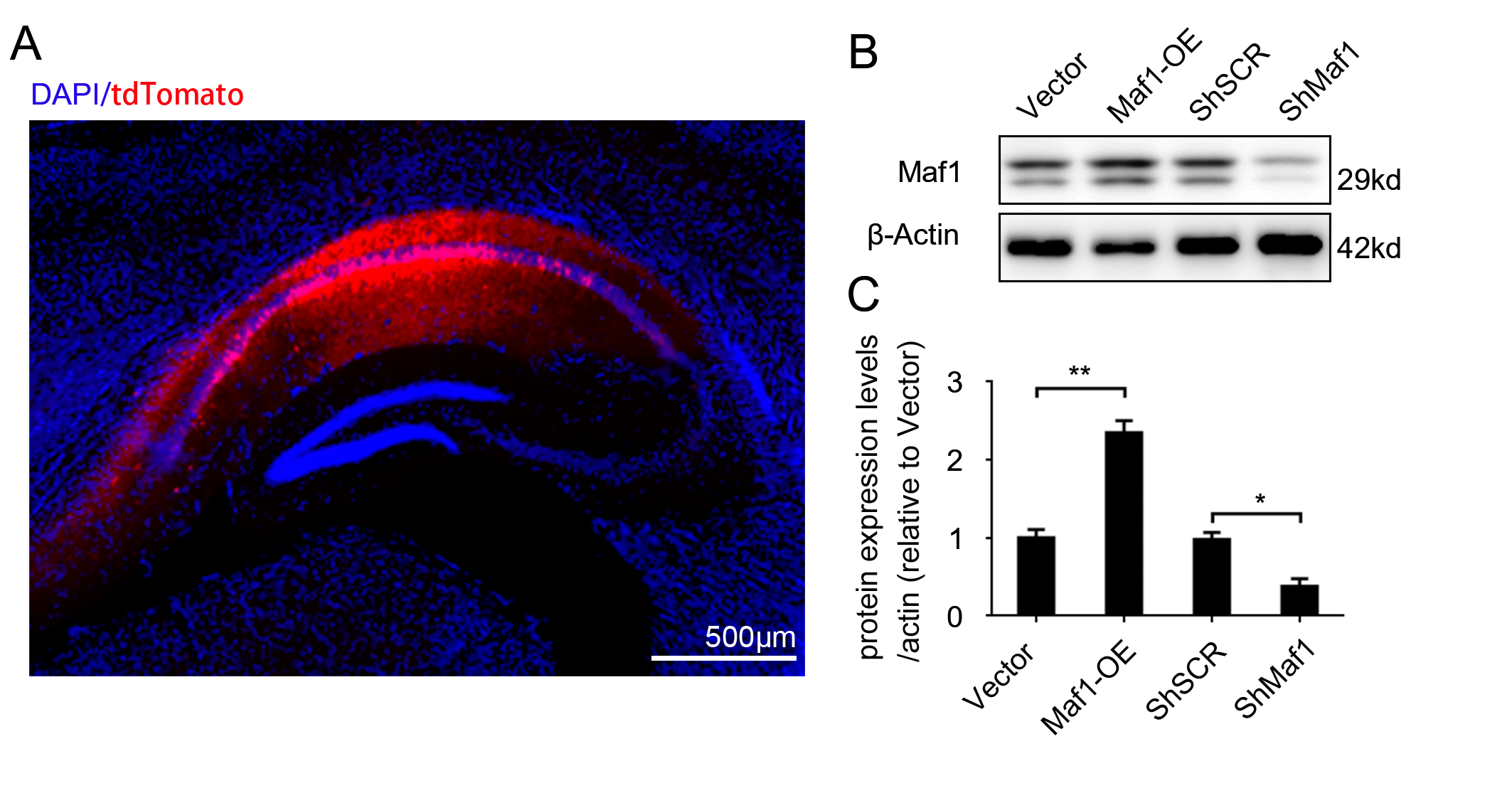

Supplement: Supplementary file 2 — Supplementary Figure 2 [file 41419_2020_2809_MOESM2_ESM.tif]
