# Supplementary material for: Maf1 regulates dendritic morphogenesis and influences learning and memory
Source: Cell Death Dis. 2020 Jul 30;11(7):606. doi: 10.1038/s41419-020-02809-y (PMC7393169; doi:10.1038/s41419-020-02809-y)
Supplement: Supplementary file 3 — Supplementary Figures Legend [file 41419_2020_2809_MOESM3_ESM.docx]

**Fig. S1 Maf1 is highly expressed in hippocampal neuron dendrites** **a, b** The expression of Maf1 in different tissues and different regions of the brain were analyzed by Western blotting. **c** The expression of Maf1 in the brain was analyzed by IF. **d** The boxes in c are shown at higher magnification in **d**. **e** Colocalization of Maf1 with Map2 in brain slices and cultured hippocampal neurons.

**Fig. S2 Overexpression of Maf1-OE-rAAV9-td-Tomato or ShMaf1-rAAV9-td-Tomato in the mouse hippocampus.** All mice were stereotaxically injected with 9.2 × 10^9^ genomes of rAAV vectors 6 weeks before analysis. **a** representative td-Tomato fluorescence in a hippocampus injected with rAAV-td-Tomato. **b, c** The expression and quantification, respectively, of immunoblots of Maf1 from hippocampal CA1 sections infected with the indicated rAAV vectors (n = 3 experiments). Error bars indicate S.E. ****, p < 0.001; **, p < 0.01; *, p <0.05.*
